# Life cycle assessment of residual lignocellulosic biomass-based jet fuel with activated carbon and lignosulfonate as co-products

**DOI:** 10.1186/s13068-018-1141-9

**Published:** 2018-05-14

**Authors:** Francesca Pierobon, Ivan L. Eastin, Indroneil Ganguly

**Affiliations:** Center for International Trade in Forest Products, School of Environmental and Forest Sciences, University of Washington, Seattle, WA 98195 USA

**Keywords:** LCA, Woody biomass, Bio-fuel, Harvest slash, Iso-paraffinic kerosene, Activated carbon, Lignosulfonate, Avoided impact, System expansion, Mass allocation

## Abstract

**Background:**

Bio-jet fuels are emerging as a valuable alternative to petroleum-based fuels for their potential for reducing greenhouse gas emissions and fossil fuel dependence. In this study, residual woody biomass from slash piles in the U.S. Pacific Northwest is used as a feedstock to produce iso-paraffinic kerosene, through the production of sugar and subsequent patented proprietary fermentation and upgrading. To enhance the economic viability and reduce the environmental impacts of iso-paraffinic kerosene, two co-products, activated carbon and lignosulfonate, are simultaneously produced within the same bio-refinery. A cradle-to-grave life cycle assessment (LCA) is performed for the residual woody biomass-based bio-jet fuel and compared against the cradle-to-grave LCA of petroleum-based jet fuel. This paper also discusses the differences in the environmental impacts of the residual biomass-based bio-jet fuel using two different approaches, mass allocation and system expansion, to partition the impacts between the bio-fuel and the co-products, which are produced in the bio-refinery.

**Results:**

The environmental assessment of biomass-based bio-jet fuel reveals an improvement along most critical environmental criteria, as compared to its petroleum-based counterpart. However, the results present significant differences in the environmental impact of biomass-based bio-jet fuel, based on the partitioning method adopted. The mass allocation approach shows a greater improvement along most of the environmental criteria, as compared to the system expansion approach. However, independent of the partitioning approach, the results of this study reveal that more than the EISA mandated 60% reduction in the global warming potential could be achieved by substituting petroleum-based jet fuel with residual woody biomass-based jet fuel. Converting residual woody biomass from slash piles into bio-jet fuel presents the additional benefit of avoiding the impacts of slash pile burning in the forest, which results in a net negative impact on ‘Carcinogenics’ and ‘Respiratory effects’, and substantial reduction in the ‘Smog’ and ‘Ecotoxicity’ impacts. The production of woody biomass-based bio-jet fuel, however, did not show any significant improvement in the ‘Acidification’ and ‘Eutrophication’ impact categories.

**Conclusions:**

The study reveals that residual woody biomass recovered from slash piles represents a more sustainable alternative to petroleum for the production of jet fuel with a lower impact on global warming and local pollution. Future research should focus on the optimization of chemical processes of the bio-refinery to reduce the impacts on the ‘Acidification’ and ‘Eutrophication’ impact categories.

## Background

Growing interest in renewable biomass-based bio-fuels for mitigating climate change and reducing fossil fuel dependence is driving the need for a better understanding of their environmental impacts [1–4]. Bio-fuels are emerging as an important class of substitutes for petroleum-based transportation fuels, dominated by ethanol produced from corn starch, generally referred to as conventional bio-fuel. Globally, bio-fuels are playing an important role in complying with policies aimed at mitigating climate change and reducing fossil fuel dependence. Given the high carbon footprint associated with air travel and rapid growth in the aviation industry, aviation bio-fuels have received a significant attention both in the private and public sectors [5].

Aviation fuel has stricter quality requirements than fuels used in road transport [5]. Requirements for bio-jet fuel are defined by the ASTM standards, which specify minimum energy density, freeze point temperature, sulfur and aromatics content, mercaptan concentration, aromatics content, fuel electrical conductivity, and flash point. To achieve these specifications, one of the pathways that have been explored consists of upgrading alcohols to drop-in bio-jet fuel, popularly known as the ‘Alcohol-to-Jet fuel’ (ATJ) pathway. Producing bio-jet fuel (i.e., iso-paraffinic kerosene or IPK) using ATJ includes upgrading alcohol using a bio-catalytic fermentation and oligomerization process. Many feedstocks have been explored to produce bio-jet fuel utilizing the ATJ pathway, including corn and corn stover, switchgrass, wheat straw, barley straw, and glucose [5]. In November of 2016, Alaska Airlines flew the first commercial flight, from SeaTac airport, using a 20% blend of bio-fuel produced via ATJ technology starting from residual woody biomass, demonstrating the feasibility of using the technology on a wood-based feedstock [6, 7].

To ensure GHG emission reductions and a sustainable bioenergy industry, sustainability criteria have been defined for bio-fuels. The EU Renewable Energy Directive requires bio-fuels to achieve a greenhouse gas emission saving of 60% for installations in which production started from 2017 onwards [8]. The US Energy Independence and Security Act (EISA) established life cycle greenhouse gas (GHG) emission reduction thresholds (against gasoline) for bio-fuels relative to a 2005 baseline [9]. As compared to the conventional bio-fuels that are required to have greenhouse gas emission reduction of about 20%, cellulosic bio-fuels are required to have a life cycle GHG emissions reduction of 60% relative to the baseline [1, 9–11]. To estimate the environmental impacts associated with the production of bio-jet fuel, a comprehensive life cycle assessment (LCA) must be performed.

LCA studies on bio-jet fuels are not uncommon in the literature. LCA studies have been conducted for bio-jet fuel produced using an ‘Oil-to-Jet fuel’ pathway, where oil is extracted from feedstocks including micro-algae, *Camelina* and *Jatropha curcas*, which is then converted to bio-jet fuel using the UOP Renewable Jet Process technique [12–14]. Budsberg et al. investigated the global warming potential of converting poplar biomass to drop-in bio-jet fuel, proposing an ‘acetogen’ fermentation pathway over an ‘ethanologen’ pathway [15]. Compared to residual woody biomass, poplar biomass and other crop-based feedstocks (e.g., micro-algae, *Camelina* and *Jatropha curcas*) present the disadvantage of additional impacts caused by plantation and land use change. A recently published comparative LCA of lignocellulosic bio-jet fuel using an ATJ process revealed that not all LCA indicators favor bio-jet fuel [16]. Some of the LCA impact categories, including ‘eutrophication’ and ‘ecotoxicity’ impacts, were worse for the bio-jet fuel. These results are consistent with some results found in the literature on the conventional bio-fuels [17, 18] and there is a general consensus on the need to extend the evaluation of the environmental impacts to impact categories other than global warming potential [2, 17–20].

To enhance the economic viability of producing drop-in fuel and reduce the overall environmental impacts, one possible solution may be to produce multiple products within the same bio-refinery [3]. When more than one product is produced from the same process, the total life cycle environmental impacts of processes can be partitioned between the product system under study and the co-products, a process known as ‘allocation’. Allocation can be done on the basis of mass, volume, or energy content of the co-products. When physical properties alone cannot be established or used, allocation may be based on the economic value of the products [21]. Because of their strong influence on LCA outcomes, allocation decisions need to have a clear, rational basis [3].

The sensitivity of impact assessments associated with the allocation choice has been an issue in LCA. The analysis of the scientific literature has shown that the LCA results with co-products heavily depend on the type of allocation used [3, 21–24]. As per ISO guidelines, it is recommended that allocation should be avoided when possible [25, 26] either through the division of the whole process into sub-processes related to co-products or by expanding the system boundaries [21].

### Objectives

This paper explores the ‘cradle-to-grave’ environmental impact of bio-jet fuel produced from residual woody biomass using an ASTM approved ATJ pathway. To undertake a comprehensive environmental assessment, this paper presents the life cycle impact assessment (LCIA) categories included in the Tool for Reduction and Assessment of Chemicals and other Environmental Impacts (TRACI), including climate change, acidification, eutrophication, smog formation, respiratory effects, carcinogenics, noncarcinogenics, and ecotoxicity. The objectives of this study are, therefore, as follows:

- perform a wood-to-wake (cradle-to-grave) life cycle assessment of lignocellulosic biomass-based bio-jet fuel with co-products;
- compare the results of the wood-to-wake (cradle-to-grave) life cycle assessment of lignocellulosic biomass-based bio-jet fuel to the well-to-wake (cradle-to-grave) life cycle assessment of petroleum-based kerosene;
- discuss the differences in the environmental impacts of residual biomass-based bio-jet fuel using two different approaches to partition the impacts between the bio-fuel and the co-products.

## Methods

### System boundary

**Fig. 1:**
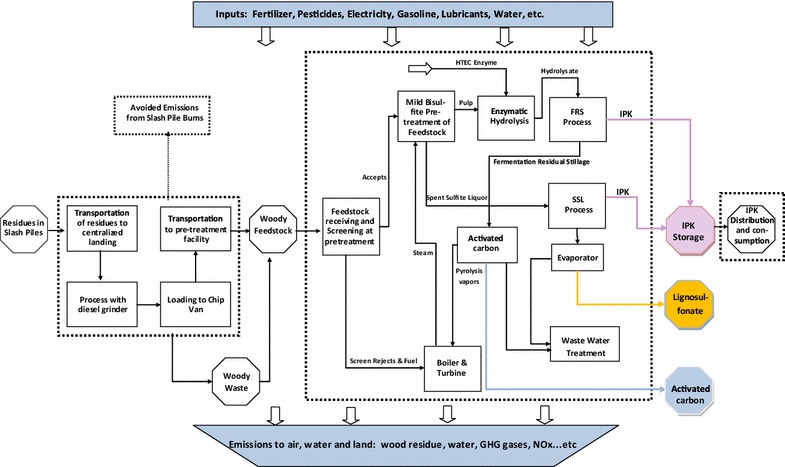
System boundary of the LCA study of bio-jet fuel with co-products

A comprehensive LCA of forest residue-based aviation fuel is performed using a ‘cradle-to-grave’ approach where ‘cradle’ is defined as forest residues collected into slash piles in the forest and ‘grave’ is defined as the combustion of the jet fuel during flight in an aircraft. As can be observed in the system boundary presented in Fig. 1, three co-products are simultaneously produced in the bio-refinery: iso-paraffinic kerosene (IPK), lignosulfonate (LS), and activated carbon (AC). For the purpose of this analysis, it is assumed that the bio-refinery is located in Grays Harbor county of Washington state. This site is identified based on its proximity to the feedstock and the available infrastructure. The area of study, which is determined by the feedstock zone, includes South Western Washington and North Western Oregon. The plant is scaled with a capacity to process 700,000 oven-dry *t* of residual woody biomass per year. The functional unit for the LCA is 1 GJ of energy for propelling an aircraft engine calculated based on a heating value for the bio-jet fuel of 43.2 MJ kg^−1^ [27]. It may be noted that, when comparing performance characteristics between fossil fuels and bio-fuels, it is important to consider the grade of substitution for the bio-fuel, which takes into account the performance of the engine when using bio-fuel instead of fossil fuel. The IPK produced by this process meets the requirements of ASTM D7566-17a for hydro-processed synthesized paraffinic kerosene, a blendstock used in jet fuel [28, 29]. As a ‘drop-in’ fuel (i.e., direct replacement), it can be blended with, or completely replace, Jet-A without necessitating any substantial modifications to engine or aircraft [30].

#### Feedstock

The cellulosic feedstock includes treetops and branches from commercial harvest operations of the timber industry in softwood forests mainly constituted of Douglas-fir, defined as post-harvest residual biomass. For undertaking an allocation of the environmental impacts associated with harvest operations, it is assumed that, on a mass basis, 39% of the above ground biomass is constituted of ‘harvest residues’ (feedstock for the bio-fuel production), excluding embedded inert material such as sand or small rocks, while the remaining 61% is logs to be used by the forest products industry.

Of all the ‘harvest residue’ left in the forest, approximately 65% of the residual biomass is accumulated into ‘slash piles’ located at the primary harvest landings [31], while the remaining 35% is scattered on the forest floor. After factoring in losses during collection, transportation, and in-wood processing, approximately 22% of the above ground biomass (i.e., 58.5% of the total harvest residuals) is delivered and utilized as feedstock for IPK production. This paper adopts the feedstock logistics base case developed by Chen et al. that is considered representative of an average scenario for the Pacific Northwest region under consideration [32, 33]. Two transportation stages are involved in this scenario. From the harvesting site, forest residues are transported to the centralized landing by a 30 m^3^ bin truck that travels an average of 1.6 km on dirt roads and an average of 5 km on gravel roads. From the central landing, residues are converted into wood chips using a diesel grinder and they are loaded into a 100 m^3^ chip van for transport to a pretreatment facility. On average, the chip vans travel 5 km on paved roads and 68.4 km on highways. In total, the biomass feedstock is transported approximately 80 km between the primary landing and the bio-refinery. The truck fuel consumptions and the distances and corresponding speeds traveled on the different road types are reported in Tables 1 and 2, respectively.

**Table 1:** Equipment characteristics. Source: ‘Modeling the Processing and Transportation Logistics of Forest Residues Using Life Cycle Assessment’ [32]

Equipment	Capacity	Productivity (1000 kg h^−1^)	Fuel consumption (l h^−1^)
Forwarder	130 kW	31.4	29.9
Excavator loader	30 kW	36.2	25.6
Grinder	560 kW	45.4	96.5
Post-grinder loader	105 kW	45.4	21.3
Bin truck	30 m^3^		
Chip van	100 m^3^

**Table 2:** Benchmark scenario for road-type-specific transportation distances. Source: ‘Modeling the Processing and Transportation Logistics of Forest Residues Using Life Cycle Assessment’ [32]

Road type, avg. speed	Dirt (8 km h^−1^)	Gravel (24 km h^−1^)	Highway (72 km h^−1^)	Total
One way haul km	1.6	10	68.4	80

##### Avoided impact from slash pile burn

Harvest slash, commonly called slash, is comprised of the leftover tree limbs, tops, and other residue that remains following logging activities. Disposing of slash by burning (a.k.a., slash burns) has traditionally been used to reduce wildfire risk and surface fuel loads after harvesting (and thinning) in western North American forests [34, 35]. The burning of woody biomass in forests (harvest slash burns and wildfires) is a major source of greenhouse gas emissions in the western US [36, 37]. Woody biomass burns emit a variety of gases and aerosols to the atmosphere, including carbon dioxide (CO_2_), carbon monoxide (CO), nitrogen oxides (NO_*x*_), volatile and semi-volatile organic compounds (VOC and SVOC), particulate matter (PM), ammonia (NH_3_), sulfur dioxide (SO_2_), and methane (CH_4_) [38]. Slash burn-related emissions of fine particulates (PM_2.5_), a highly potent air pollutant, have been linked to a broad range of human health issues, including an increase in the chance of contracting bronchitis and, in some cases, death [39]. Recovering post-harvest residual woody biomass to produce bio-jet fuel results in avoiding the impacts associated with its burning. In this study, two scenarios are considered for the avoided impacts of prescribed burns, which assume, respectively, that 50% (base case) and 100% of the biomass is recovered from slash piles to produce bio-jet fuel. In the 50% burn scenario, we assume that 50% of the biomass in slash piles would have been disposed by burning, had we not collected it for bio-conversion. The remaining biomass in slash piles is assumed to be collected, chipped, and sold as hog fuel or pulpwood. Recovering residual biomass avoids the emissions generated during prescribed burns. Hence, based on ISO 14040-44 standards [25, 26], the avoided environmental impacts of slash pile burning in the region were incorporated in the LCA as a credit, corresponding to avoiding burning, respectively, 50 and 100% of the biomass in slash piles in the 50% scenario and 100% scenario. The data for slash pile burn-related emissions were extracted from the National Energy Technology Laboratory Life Cycle Inventory [40].

#### Feedstock handling at the bio-refinery

Based on the previously described production scale, the total feedstock entering the bio-refinery is 91,373 oven-dry kg h^−1^ of residuals woody biomass. Before the feedstock enters the pretreatment facility, it is necessary to ensure that its composition and dimensional characteristics are constant over time. For this reason, the feedstock is sent to a feedstock handling department, which receives forest residuals and processes them into the appropriate size for pretreatment (3–19 mm). The department includes systems to weigh, sample, record data, and unload truckloads of forest residuals [41]. A screen is used to separate fines from the rest of the feedstock. Fines, which represent 9% of the total feedstock on a mass basis, are collected and sent to the boiler for the production of steam to be used in the plant, while the remaining 91% of the feedstock is sent for pretreatment.

#### Pretreatment

A major challenge to the production of bio-fuel from biomass is that the fermentable sugars are trapped inside the lignocellulose. This material is resistant to degradation and is responsible for the stability and structural integrity of plant cell walls. Pretreatment is a necessary process in the conversion of biomass to bio-fuel, as the breakdown of the biomass in this stage facilitates the downstream enzymatic hydrolysis. During the pretreatment process, most of the hemicellulose carbohydrates are converted to soluble sugars (xylose, mannose, arabinose, and glucose).

Woody biomass has a tough and strong physical structure and a high lignin content that makes it very recalcitrant to microbial destruction [42]. Given its strong recalcitrance, only a few pretreatment technologies have proven to be applicable to woody biomass [42]. This study uses the ‘Sulfite Pretreatment to Overcome Recalcitrant of Lignocellulose’ (SPORL) method, which uses calcium bisulfite (Ca(HSO_3_)_2_) [43, 44].

The feedstock is sent to a reactor where sulfur and calcium carbonate are added. Sulfur is burned in a furnace at a 10:1 air-to-sulfur ratio at 1300 °C to form SO_2_. Calcium carbonate is mixed with water and combined with the SO_2_ in the acid preparation absorption column. Biomass is treated at 145 °C for 4 h, during which the hemicellulose bonds break and the pretreated pulp is prepared to be subsequently treated by enzymatic hydrolysis. The pretreatment process produces two outputs: pulp, primarily consisting of insoluble solids which is sent to enzymatic hydrolysis, and spent sulfite liquor (SSL), primarily consisting of sugars, extractives, and lignosulfonate which are sent directly to the fermentation and upgrading phase.

#### Enzyme production and enzymatic hydrolysis

During enzymatic hydrolysis, cellulase enzymes (catalytic proteins) are used to break down the cellulose fibers contained in the pretreated pulp into monomeric sugars. For the enzyme production, glucose, lime, ammonia, corn steep liquor, and sulfur dioxide are combined in a fermentator to produce a culture of *Trichoderma reesei* (filamentous fungi strain). The fungal culture is inoculated into an enzyme production reactor and is induced with sophorose, which begins the enzyme production. The produced enzyme is separated from the fungal biomass through filtration. Energy is required for the fungal biomass separation and subsequent concentration. The enzymes are then mixed with the pulp stream from pretreatment, cooled from 80 to 50 °C, and are pH adjusted from 1.8 to 5.0 by adding lime. During enzymatic hydrolysis, the pretreated pulp is treated for 72 h at 50 °C. Following enzymatic hydrolysis, the majority of the macromolecules of cellulose and hemicellulose are converted into fermentable sugars and the hydrolysate is sent to the fermentation process.

#### Fermentation and upgrading

The hydrolysate, obtained from enzymatic hydrolysis, and the spent sulfite liquor (SSL), obtained from pretreatment, are converted to alcohol and subsequently to iso-paraffinic kerosene (IPK) by means of a proprietary patented fermentation and upgrading process [29]. Given the different compositions of hydrolysate and SSL, the hydrolysate is characterized by a high content of insoluble lignin and solids, while the SSL mostly contains sugars and soluble lignin. The two streams are treated in different units to maximize the conversion yield. The fermentation and upgrading process consists of three major phases: (1) fermentation of sugar into iso-butanol; (2) separation and purification of iso-butanol; (3) dehydration, oligomerization, and hydrogenation of iso-butanol into IPK. Sugars are converted into iso-butanol using a combination of specifically engineered enzymes. The fermentation process requires 48 h at 34 °C and pH 4.3. The iso-butanol is then separated, dehydrated, and converted to butylene, which is subsequently upgraded to IPK. Based on the modeled production scale, the quantities of IPK obtained from hydrolysate and SSL are, respectively, 10,213 and 1989 kg h^−1^, for a total IPK production of 12,202 kg h^−1^. The residual portion of hydrolysate that is not converted to iso-butanol (e.g., fermentation residual stillage (FRS) which is rich in insoluble solids and lignin is recovered and sent to the pyrolysis unit. The residual portion of SSL [e.g., lignosulfonate (LS)] is mainly constituted of unconverted sugars and soluble solids, and is sent to the upgrading process.

#### Upgrading lignosulfonate

The lignosulfonate and the residual output of the SSL fermentation and upgrading process is sent to an evaporation unit, where a series of vapor recompression evaporators are used to reduce its water content to a concentration of 50% solids. The 50% concentrated lignosulfonate (LS 50%) is produced at a rate of 21,192 kg h^−1^. The evaporator condensate is discharged directly into the wastewater treatment facility. The LS 50% has an important commercial value, as it is used by the concrete industry to either improve the workability of the concrete mix and/or reduce the water/cement ratio and, for this reason, is considered a co-product.

#### Pyrolysis unit

The FRS, a residual of the fermentation of hydrolysate, is dewatered though a belt press and the excess water is discharged to the wastewater treatment facility. The dry FRS is placed into a rotary kiln reactor for pyrolysis, at 700 °C for 1 h. The reactor is fed with a nitrogen carrier gas at a 1:1 nitrogen-to-solid mass ratio. The pyrolysis process produces 40% (w/w) biochar and 60% pyrolysis vapors. The biochar is activated, by reacting it with excess CO_2_ at 700 °C for 1 h. The activation reaction generates a yield of 55%, which results in a 22.5% (w/w) yield of activated carbon (AC) based on input FRS, for a total AC production rate of 6798 kg h^−1^. Due to its high degree of microporosity, AC is a commercially valuable product used for its adsorption properties and, therefore, is considered a co-product. AC is widely employed in gas purification, decaffeination, gold purification, metal extraction, water purification, medicine, sewage treatment, air filters in gas masks and respirators, filters in compressed air, and many other applications.

#### Wastewater treatment

The wastewater treatment receives the water output streams from the Fermentation and Upgrading unit, Lignosulfonate Upgrading unit, and the Pyrolysis unit. The residual portion of solids that is not converted in the Pyrolysis unit is also sent to the wastewater treatment facility. The wastewater treatment facility includes the following units: aerobic and anaerobic treatment, membrane bio-reactor, and reverse osmosis. The aerobic treatment converts 86% of the biomass, and 74% of the remaining biomass is converted in an anaerobic treatment. The fully digested material is sent to a membrane reactor for clarification, removing additional chemical oxygen demand (COD) and colloidal particles. The total digestion yield calculated as the ratio of digested biomass to the total degradable biomass is 99.95%. The treated water is then sent through a reverse osmosis membrane system for salt removal. The wastewater plant treats about 556,326 kg h^−1^ of water and discharges about 7056 kg h^−1^ of treated water to the environment.

The wastewater treatment facility produces about 16,625 kg h^−1^ of wet sludge, which is dewatered and sent to the boiler. In addition, the anaerobic treatment process produces 61.6 kg h^−1^ of methane and other combustible gases, which is captured and sent to the boiler. The use of sludge and biogas in the multi-fuel boiler reduces the hog fuel consumption by 4082 oven-dry kg h^−1^. Approximately 520,882 kg h^−1^ of water is recycled back into the production system.

#### Boiler

The multi-fuel boiler is fed with the fines, the small-sized fraction of woody biomass that is rejected from the handling department, hog fuel, pyrolysis vapors, dewatered sludge, and the biogas recovered from the wastewater treatment process. The boiler efficiency, defined as the percentage of the feed heating value that is converted to steam heat on a higher heating value (HHV) basis, is assumed to be 80% [45]. A total of 210,367 kg h^−1^ of steam are produced from the boiler and distributed to the bio-refinery departments. The condensate system returns the steam condensate from the plant into a tank which is reused as boiler feed water. The boiler exhaust gas contains ash which is collected in a baghouse and sent to the landfill. The fine percent ash is 1.97% and the hog fuel percent ash is 6.8%.

#### Transportation of IPK to SeaTac airport and combustion in an aircraft engine

The IPK is transported from the production facility to Seattle-Tacoma International Airport (SeaTac) in a diesel truck (the traveled distance is 33.7 km) and distributed to the final user. IPK is burned in an aircraft engine for transportation in a commercial passenger flight. A heating value of 43.1 MJ kg^−1^ is assumed for the petroleum-based jet fuel and of 43.2 MJ kg^−1^ for the bio-jet fuel [27].

### Data

This paper uses a combination of primary and secondary data, from a number of sources, to conduct the LCA analysis. For the feedstock logistics component, this paper adopts the feedstock logistics base case for Pacific Northwest (PNW) developed by Chen et al. [32]. The NETL Life Cycle Inventory Data [40] are utilized for the emissions of slash piles.

For the pretreatment units, the model uses the SPORL process, which was specifically developed to prepare cellulosic feedstock for enzymatic hydrolysis [44]. The Gevo, Inc. patented GIFT^®^ process is used to model the bio-catalytic conversion of fermentable sugars to iso-butanol (iBuOH) and the upgrading process [29]. The proprietary bio-catalytic conversion data, necessary for conducting the LCA, were provided by Gevo. The wastewater treatment and the boiler systems were modeled based on the reactions stoichiometry and yields used in the NREL model for converting lignocellulosic biomass into ethanol [45].

Integrating all the sub-processes within the bio-refinery, a detailed chemical process simulation was performed using Aspen Plus 8 software for the simulation of chemical processes and the scale up from laboratory to industrial scale [46]. The results of the chemical process simulation provided high-level mass and energy balances, as well as a list of all the material and energy input and output flows of the bio-refinery, for use in the LCA. Data for all the input and output flows from the technosphere and from nature, as well as any emissions to air, water, or soil or solid waste produced within the bio-refinery were collected and included in the LCA. Major mass and energy inputs and output data, in aggregated form, for the bio-refinery units are summarized in Table 3. The complete set of primary data used in the LCA is reported in ‘Process Design and Economics for Biochemical Conversion of Softwood Lignocellulosic Biomass to Iso-paraffinic Kerosene and Lignin Co-products’ [46]. Secondary data for the input and output flows were obtained from the Ecoinvent database for the processes of transportation of IPK to SeaTac airport and combustion in an aircraft engine [47].

**Table 3:** Aggregated mass and energy inputs and outputs for the bio-refinery units. Source: adapted from ‘Process Design and Economics for Biochemical Conversion of Softwood Lignocellulosic Biomass to Iso-paraffinic Kerosene and Lignin Co-products’ [46]

Feedstock preparation and pretreatment		Enzymatic hydrolysis		Fermentation and upgrading, pyrolysis unit and lignosulfonate concentration		Wastewater treatment		Boiler	
*Inputs (kg h* ^*−1*^ *)*	*450,018.2*	*Inputs (kg h* ^*−1*^ *)*	*404,513.7*	*Inputs (kg h* ^*−1*^ *)*	*737,715.3*	*Inputs (kg h* ^*−1*^ *)*	*556,326.6*	*Inputs (kg h* ^*−1*^ *)*	*342,686.6*
Forest residual	91,372.9	Pretreated pulp	65,507.8	Hydrolysate	69,662.7	Fermentation residual stillage	8308.0	Hog fuel	31,297.9
Sulfur	2993.7	Corn steep liquor	199.6	Spent sulfite liquor	24,604.7	Spent sulfite liquor condensate	1886.0	Forest residual fines	8223.6
Calcium carbonate	2948.4	Glucose	3401.9	Proprietary inputs [41]	29,057.1	Chemicals	43.6	Pyrolysis vapors	28,503.7
Sodium hydroxide	113.4	Lime	471.7	Pyrolysis carrier gas	27,215.5	Water^a^	546,088.9		
Water^a^	60,920.9	NH_3_	136.1	Water^a^	587,175.3			Biogas	3527.6
Process water^b^	291,669.0	SO_2_	18.1					Sludge	5681.5
		HTEC enzyme	263.1					Combustion air	27,215.5
		Water^a^	129,319.2					Water^a^	27,869.8
		Process water**	205,196.1					Steam condensate	210,367.1
*Outputs (kg h* ^*−1*^ *)*	*450,022.5*	*Outputs (kg h* ^*−1*^ *)*	*404,514.4*	*Outputs (kg h* ^*−1*^ *)*	*737,715.1*	*Outputs (kg h* ^*−1*^ *)*	*556,326.1*	*Outputs (kg h* ^*−1*^ *)*	*342,686.7*
Pretreated pulp	65,507.8	Hydrolysate	69,662.7	IPK	12,201.6	Waste water solids	11.1	Steam	210,367.1
Spent sulfite liquor	24,604.7	Saccharification vent	1711.7	Lignosulfonate	21,191.8	Biogas	3527.6	Waste	2421.8
Pretreatment combined vent	850.9	Water^a^	333,140.0	Activated carbon	6797.5	Sludge	5681.5	Emissions to air	102,028.0
Forest residual fines	8223.6			Fermentation residual stillage	8308.0	Emissions to air	1017.0	Water^a^	27,869.8
Water^a^	350,835.6			Spent sulfite liquor condensate	1886.0	Water^a^	25,207.4		
				Pyrolysis vapors	28,503.7	Recycling water^b^	520,881.5		
				Emissions to air	16,104.3				
				Water^a^	642,722.2				
*Energy inputs*									
Steam (kg h^−1^)	71,939.7	Steam (kg h^−1^)	444.5	Steam (kg h^−1^)	137,982.8	Steam (kg h^−1^)	0.0	Steam (kg h^−1^)	0.0
Electricity (MWh)	4.7	Electricity (MWh)	0.2	Electricity (MWh)	37.5	Electricity (MWh)	9.9	Electricity (MWh)	2.0

^a^Total water content of the inputs/outputs

^b^Process water added to the system (recycling water from wastewater treatment)

### Environmental impact assessment

The life cycle assessment (LCA) method, following ISO 14040-14044 standards [25, 26], was used to estimate the overall net environmental impact associated with producing bio-jet fuel from recovered residual woody biomass. The life cycle environmental impacts were assessed using the Tool for Reduction and Assessment of Chemicals and Other Environmental Impacts (TRACI 2.1) [48]. The following impact categories were included: global warming, smog, acidification, eutrophication, carcinogenics, noncarcinogenics, respiratory effects, and ecotoxicity. The life cycle inventory analysis and impact assessments were conducted using SimaPro 8. As per the IPCC Fifth Assessment Report, this paper reports the 100 year impacts for the global warming potential [49]. The USLCI database was used to model the impacts of each input and output flow. Only for the input and output flows, where no USLCI database was available, the Ecoinvent database was used. Utilizing a ‘woods-to-wake’ (WoTW) LCA approach, which is comparable to a well-to-wake (WTW) LCA for petroleum-based aviation fuel, the environmental implications of feedstock recovery, production, and utilization of residual woody biomass-based bio-jet fuel were assessed. A comparative assessment of the environmental implications of substituting petroleum-based jet fuel with that of residual woody biomass-based bio-jet fuel was also conducted. Though the US Department of Energy’s Greenhouse gases, Regulated Emissions and Energy use in Transportation (GREET) software has a model associated with aircraft operations for the US [50], it focuses only on greenhouse gas emissions and the global warming potential (GWP) impact category and does not provide the necessary data associated with the nonGWP LCIA categories as identified in TRACI. The processes associated with fossil fuel-based bio-jet fuel were modeled using Simapro 8.1. For the GWP impact category, the results were compared against the baseline lifecycle greenhouse gas emissions, as defined in the Energy Independence and Security Act of 2007 [11] and as developed by the US Department of Energy [51].

### Allocation approach for addressing multiple products

As was introduced in the system boundary section, the co-products simultaneously produced in the bio-refinery process are modeled in this paper. The methodology adopted for allocating the environmental impacts of the bio-refinery among the co-products can significantly affect the results. Allocation is defined as: partitioning the input and/or output flows of a process to the product system under study [25, 26]. As per ISO standards, it is recommended that allocation should be avoided when possible, either by expanding the system boundary (a.k.a. system expansion) or by dividing the process into sub-processes (a.k.a. system reduction). If it cannot be avoided, allocation can be undertaken on the basis of physical properties of the co-products (e.g., mass or energy content of the output) or on the basis of nonphysical properties (e.g., primarily economic value) of the products. The ISO guidelines indicate that the applicability of each allocation procedure should be evaluated on a case-by-case basis [3, 26]. Among the allocation procedures, the mass and energy allocations can be applied where the products are used for their mass and energy content purposes, respectively [52]. When physical properties alone cannot be established or used, allocation may be based on the economic value of the products [21]. However, the economic allocation approach is only applicable if the prices of products are well established or can be predicted with high confidence [52].

In this study, two out of three co-products, activated carbon and lignosulfonate, are nonenergy products and IPK is an energy product. In the case of a mixture of energy and nonenergy products, energy allocation can be problematic [52]. Economic allocation could be a viable alternative when products are defined by different physical properties. However, since the production of activated carbon, lignosulfonate, and IPK, at the proposed scale, is unprecedented in the PNW region, the market prices for these products cannot be established with high confidence. Any attempt to assign market prices to these products will be based on broad assumptions, which can lead to uncertainties associated with the modeling outputs. Allocating energy and emission burdens based on such uncertain prices may lead to misleading conclusions from the LCA results. Therefore, reducing the spectrum of the allocation approaches to the ones that are applicable to the study, the ‘mass allocation’ and the ‘system expansion’ approaches were chosen as the most viable alternatives.

#### Mass allocation approach

**Fig. 2:**
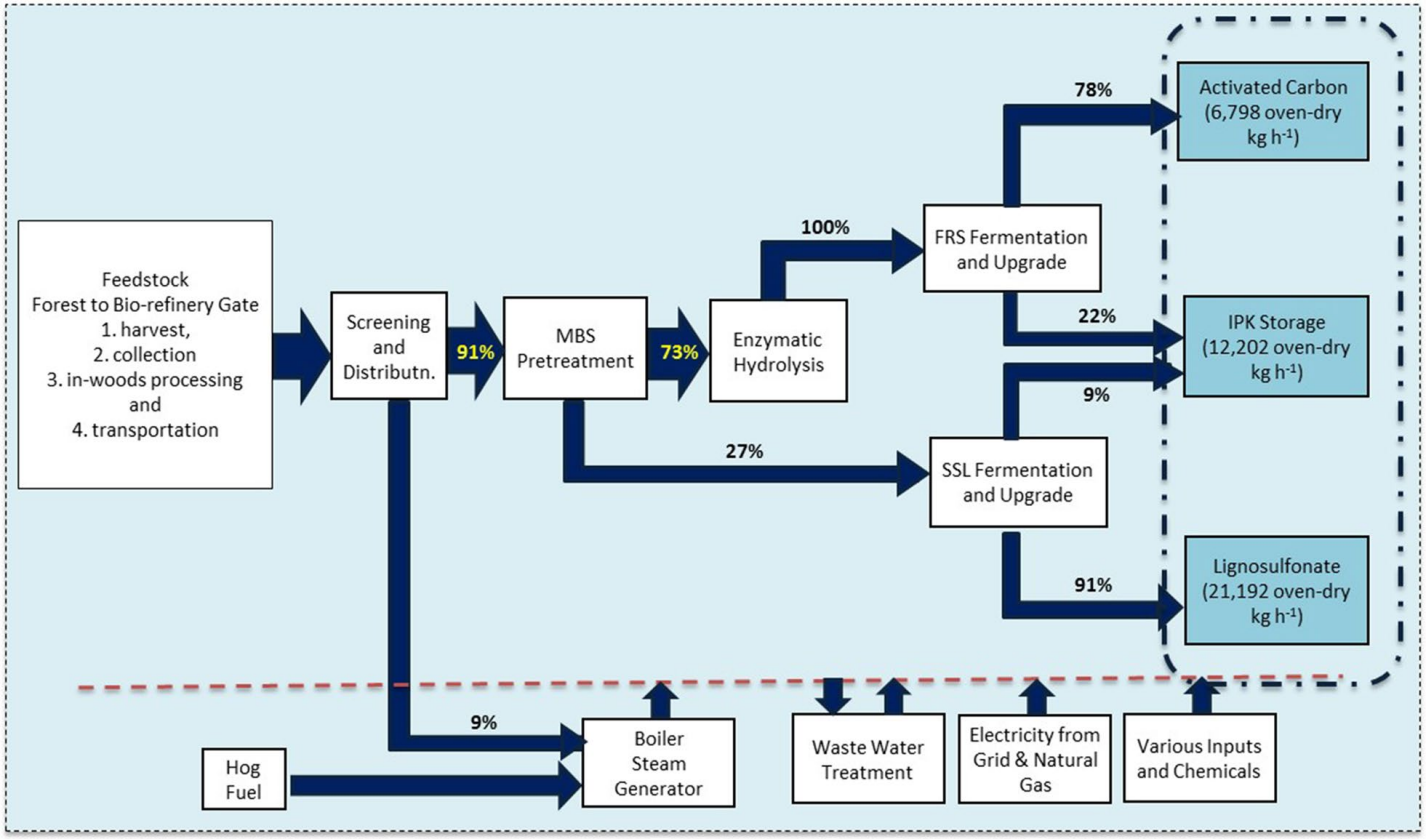
Representation of the LCA system boundary using mass allocation

Using ‘mass allocation’, the life cycle environmental impacts are allocated among co-products according to their mass output shares. This allocation method is based on the assumption that the environmental impacts are related to the mass flows associated with the production process. The allocation percentages applied to different processes, based on mass flows and the system boundary, are presented in Fig. 2.

In this case, after the feedstock enters the bio-refinery gate, 91% of the impacts of feedstock handling and screening are attributed to the biomass entering the pretreatment unit and 9% are attributed to fines. The impacts of the pretreatment unit are allocated 73% to pulp (which continues to enzymatic hydrolysis) and 27% to SSL (which is sent to fermentation and upgrading). The enzymatic hydrolysis impacts are 100% attributed to the hydrolysate (which is sent to fermentation and upgrading). The impacts of fermentation and upgrading of SSL are allocated between activated carbon (78%) and IPK (22%) and the impacts produced from fermentation and upgrading of FRS are allocated between IPK (9%) and lignosulfonate (91%). Wastewater treatment impacts are allocated between IPK (31%), activated carbon (17%), and lignosulfonate (52%), based on their production yields. It should be noted that, considering how the integrated processes are modeled, downstream inputs/outputs contain all the impacts of the upstream inputs/outputs according to the allocation ratios applied.

#### System expansion approach

**Fig. 3:**
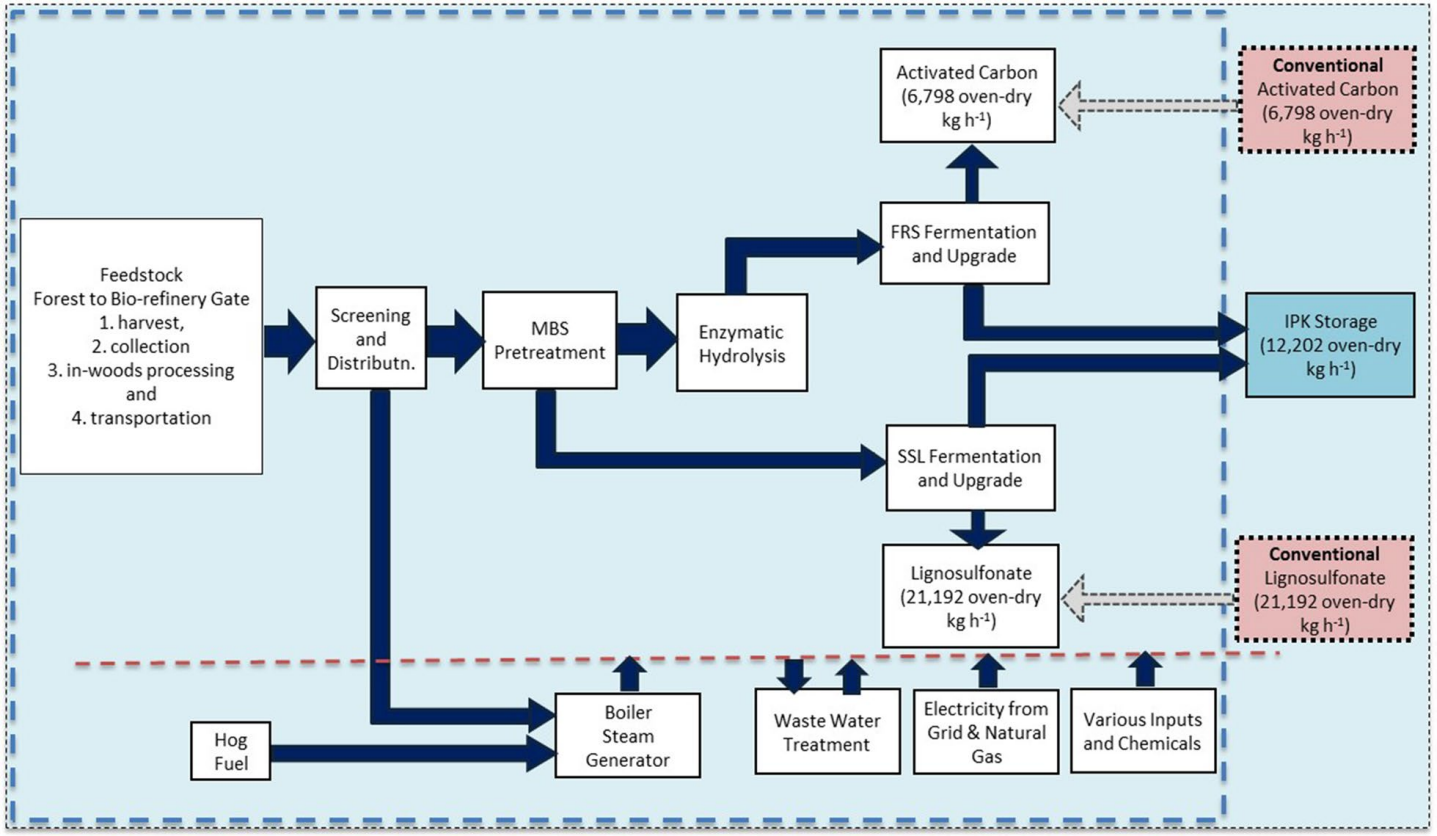
Representation of the LCA system boundary using system expansion

The ‘system expansion’ or ‘displacement method’ is identified as the preferred procedure in the ISO 14044 standards [26]. System expansion is also advocated as the recommended approach by the UK Renewable Fuels Association [53] and the US Environmental Protection Agency [54]. According to the system expansion approach, the life cycle environmental impacts of the bio-refinery are 100% attributed to the main product of study. To account for the co-products, the impacts associated with the production of the same quantities of co-products using the conventional processes are subtracted from the total. A representation of the system boundary using system expansion is shown in Fig. 3.

The impacts of the feedstock (including harvesting, collection, in-wood processing, and transportation) and those of the bio-refinery (including handling, pretreatment, enzymatic hydrolysis, fermentation and upgrade, pyrolysis unit, lignosulfonate concentration, boiler, and wastewater treatment and utilities) are 100% attributed to IPK (main product). Since activated carbon and lignosulfonate are simultaneously produced in the bio-refinery, the life cycle impacts of producing the same quantities of the conventional activated carbon and conventional lignosulfonate (respectively, 6798 and 21,192 kg h^−1^), are subtracted from the total IPK impact. Life cycle impacts of the conventional activated carbon and lignosulfonate were extracted from Gabi professional database [55] and the environmental product declaration of lignosulfonate, respectively [56].

## Results and discussion

### Contribution analysis

The contribution analysis identifies the unit processes contribution to the overall LCA. Contribution analysis is especially critical in identifying the high contributing units for the relevant LCIA factors and is frequently used as a process update decision tool. In the following sections, all the units of the woods-to-wake (WoTW) system boundary are presented, for both mass and system expansion allocation alternatives.

#### Contribution analysis of IPK based on mass allocation

**Fig. 4:**
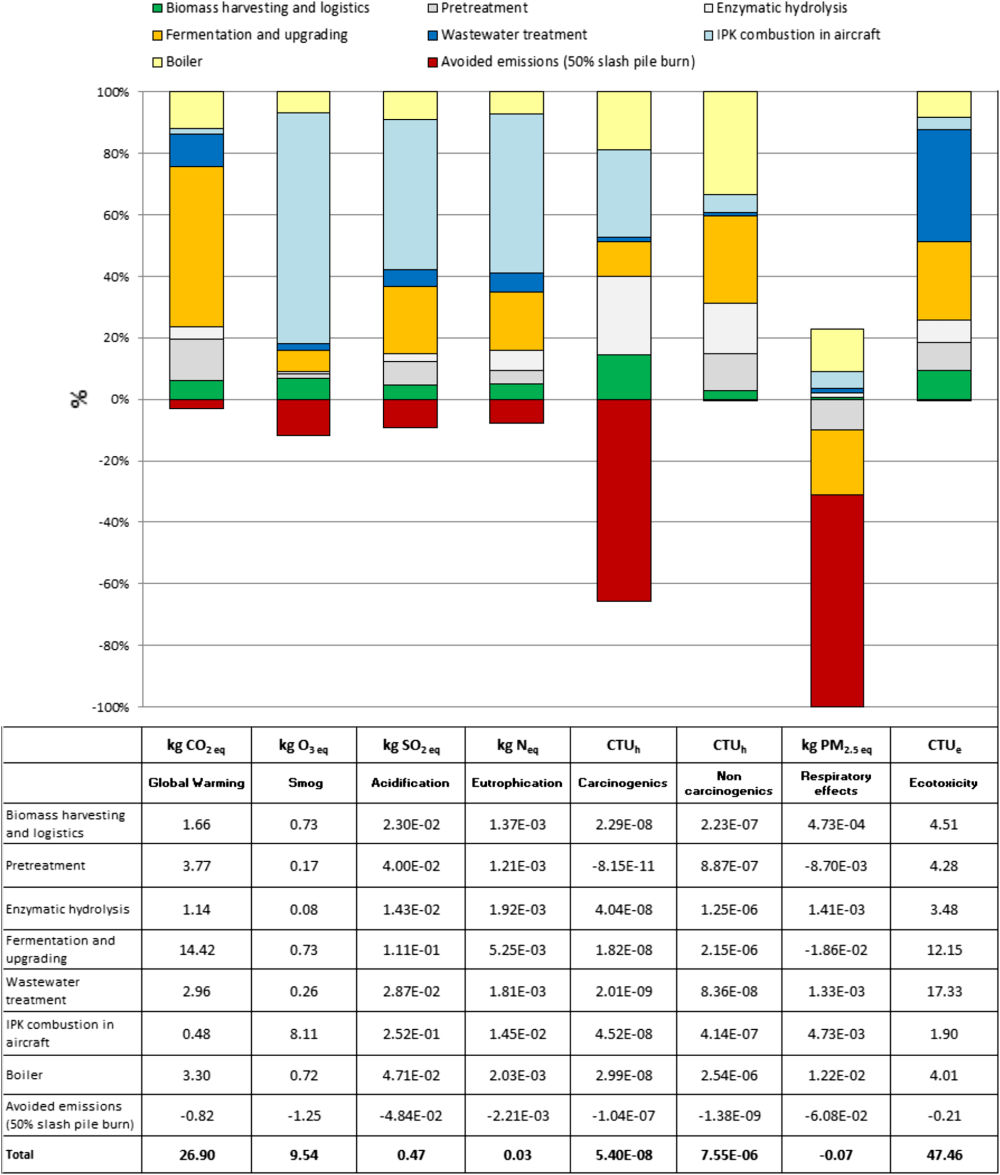
Results of the contribution analysis using mass allocation (functional unit: 1 GJ)

The results of the contribution analysis using mass allocation are presented in Fig. 4. Each of the units within the WoTW system boundary may have a favorable or unfavorable effect on the corresponding impact categories. The top section of the graph shows the positive contributions (unfavorable environmental impacts) to the LCA, while the bottom section shows the negative contributions (favorable environmental impacts) to the LCA.

Using a mass allocation-based LCA, the main contributors to global warming potential (GWP) are: fermentation and upgrading (52.0%), boiler (11.9%), pretreatment (13.6%), wastewater treatment (10.7%), biomass harvesting and logistics (6.0%), enzymatic hydrolysis (4.1%), and IPK combustion in an aircraft (1.7%). The avoided slash pile burn helps to reduce the GWP impact by only 3.0%. The ‘Smog’, ‘Acidification’, ‘Eutrophication’, and ‘Carcinogenics’ impact categories are dominated by the IPK combustion in an aircraft. More precisely, the IPK combustion in an aircraft contributes to 75.1% of the ‘Smog’ impact, biomass harvesting, and logistics to 6.8%, boiler emissions to 6.7%, and fermentation and upgrading to 6.8%, with the remaining processes contributing to 4.6%. The main processes contributing to the ‘Acidification’ impact are the IPK combustion in an aircraft (48.8%), fermentation and upgrading (21.6%), and boiler emissions (9.1%), with the remaining processes contributing to 20.5%. The same processes which cause ‘Acidification’ are also the main contributors to the ‘Eutrophication’ impact, and include: IPK combustion in an aircraft (51.5%), fermentation and upgrading (18.7%), and boiler emissions (7.2%), with the remaining processes contributing to 22.6%. The ‘Carcinogenics’ impact is caused by IPK combustion in an aircraft (28.5%), followed by enzymatic hydrolysis (25.5%) and boiler emissions (18.9%), with the remaining processes contributing to 27.1%.

The boiler is the main contributor to the ‘Non carcinogenics’ impact (33.6%), while the wastewater treatment facility is the main contributor to the ‘Ecotoxicity’ impact (36.4%), with the remaining processes contributing to 30.0%. The complete results of the contribution analysis of the LCA based on mass allocation are presented in Fig. 4.

#### Contribution analysis of IPK based on system expansion

**Fig. 5:**
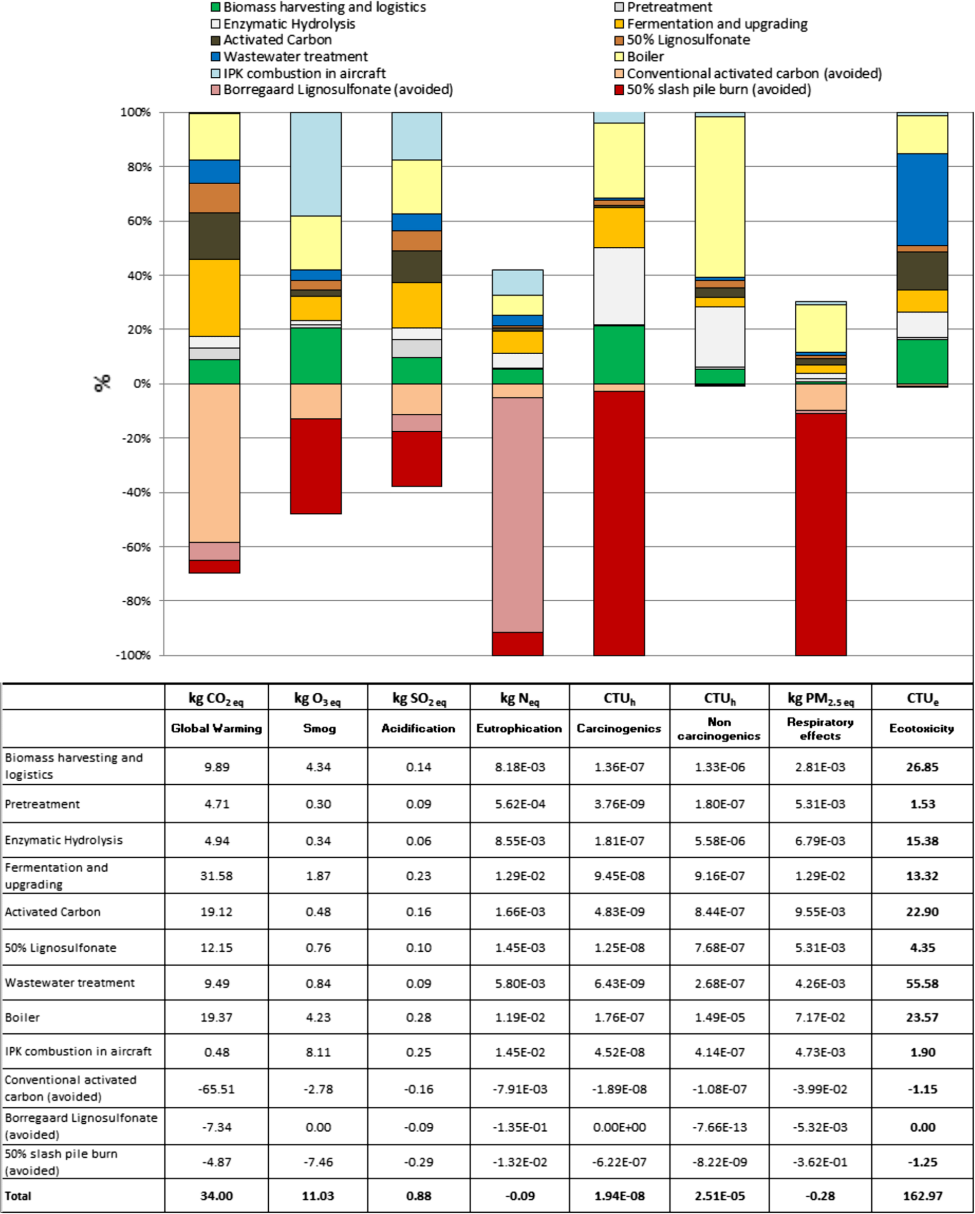
Results of the contribution analysis using system expansion (functional unit: 1 GJ)

The results of the contribution analysis using the system expansion approach are represented in Fig. 5. The key environmental benefits associated with the production of residual biomass-based bio-jet fuel are the avoided emissions from not burning the residual slash pile (represented in ‘red’) and the avoided impacts associated with the production of the two co-products, activated carbon (represented in ‘orange’) and lignosulfonate (represented in ‘pink’). In the system expansion approach, when co-products are simultaneously produced within the same process, a credit is given for the avoided impacts associated with their production using the conventional processes.

As shown in Fig. 5, the production of activated carbon using the conventional processes produces significant impacts on the GWP and impact on the ‘Smog’, ‘Acidification’, and ‘Respiratory effects’ impact categories. The production of lignosulfonate using the conventional processes produces a significant impact on the ‘Ozone depletion’ and ‘Eutrophication’ impact categories.

According to the system expansion method, the main contributors to GWP are: fermentation and upgrading (28.3%), followed by boiler (17.3%), activated carbon (17.1%), 50% lignosulfonate (10.9%), biomass harvesting and logistics (8.8%), wastewater treatment (8.5%), enzymatic hydrolysis (4.4%), pretreatment (4.2%), and IPK combustion in an aircraft (0.4%). A significant negative contribution to GWP is due to the avoided impact of producing activated carbon. Compared to the GWP results using mass allocation, using system expansion, the results show different % contributions. Using system expansion, the impact of lignosulfonate upgrading and activated carbon production, as well as the impacts of the boiler and of the wastewater treatment are entirely attributed to IPK. The inclusion of these contributions significantly affects how the impacts of processes are repartitioned relative to the total. Regarding the ‘Smog’ impact, using system expansion, the main contributors are the IPK combustion in an aircraft (38.1%), biomass harvesting and logistics (20.4%), and boiler (19.9%), with the remaining processes contributing to 21.6%.

For the ‘Acidification’ impact, the main contributors are represented by the boiler (19.6%), the IPK combustion in an aircraft (17.8%), and the fermentation and upgrading (16.5%), with the remaining processes contributing to 46.1%. The ‘Eutrophication’ impact is mainly related to the IPK combustion (9.3%), fermentation and upgrading (8.3%), and boiler (7.7%). However, the impact on ‘Eutrophication’ of the whole IPK production is more than offset by the avoided impact of Borregaard lignosulfonate, producing an overall net negative result.

The impact on ‘Carcinogenics’ is almost completely offset by the avoided impact of slash pile burning. Even more important is the effect of the avoided impact of slash pile burning on the ‘Respiratory effects’ impact, which produces an overall net negative impact. No avoided impact contributions have been accounted for in the impact categories ‘Ecotoxicity’ and ‘Non carcinogenics’. While the ‘Ecotoxicity’ impact is mainly related to the wastewater treatment (33.6%), the ‘Non carcinogenics’ impact is mainly due to the boiler emissions, which contribute to 59.1% of the total. The complete result set of the contribution analysis of the LCA based on system expansion is reported in Fig. 5.

### Comparative LCA

The results of the LCA of bio-jet fuel based on mass allocation and system expansion were compared against the LCA of petro-jet fuel. Both scenarios for the slash pile burn, as described in par. 2.1.1.1, were considered, which assume, respectively, that 50 and 100% of the biomass are recovered from slash piles to produce bio-jet fuel. The two scenarios are referred to as ‘50% scenario’ and ‘100% scenario’.

#### Comparison of fossil fuel-based kerosene and IPK based on mass allocation

**Fig. 6:**
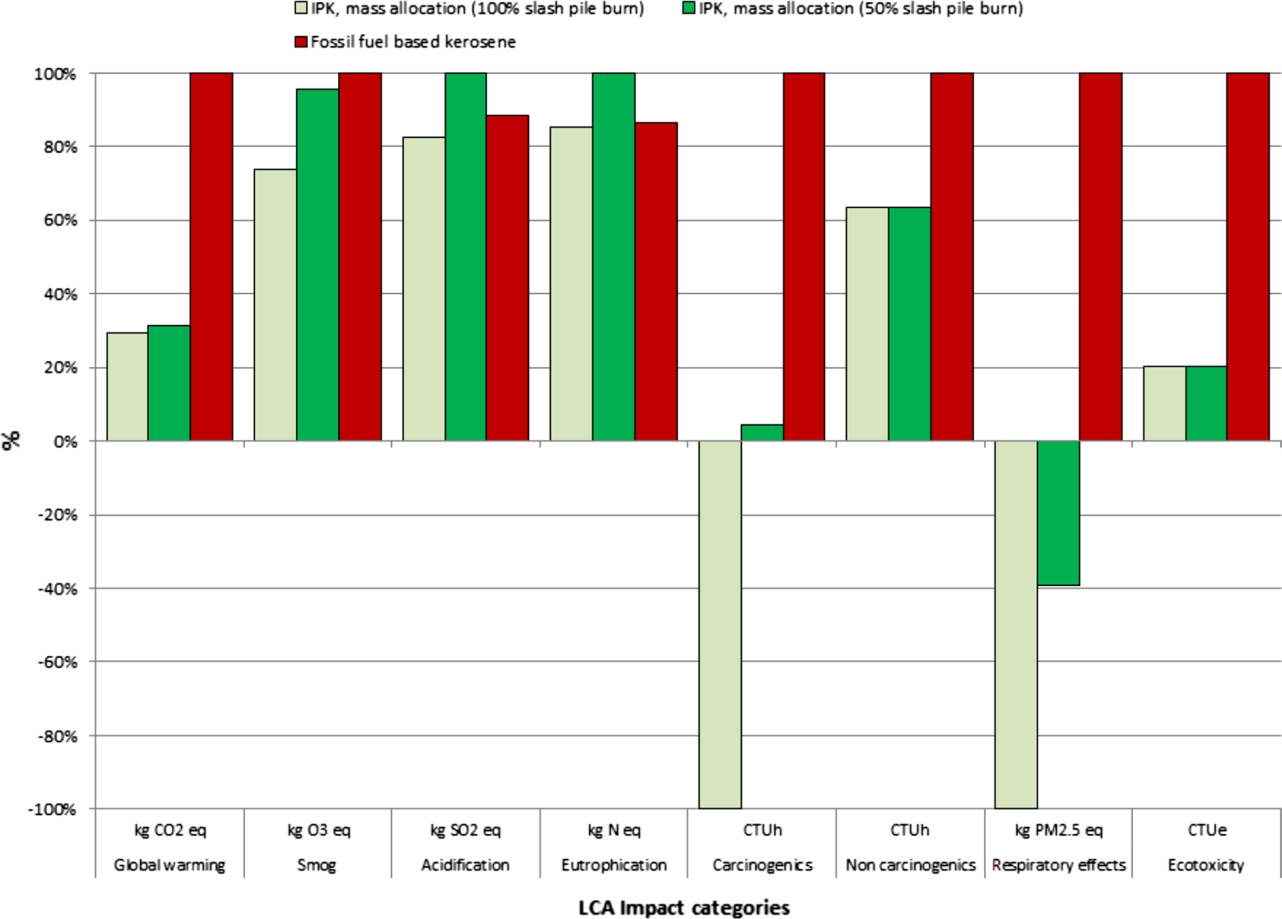
Results of comparative LCA of fossil fuel-based kerosene and IPK based on mass allocation (functional unit: 1 GJ)

The ‘cradle-to-grave’ comparative analysis of petro-jet and bio-jet fuel based on mass allocation reveals that a more than 60% reduction in the global warming potential, as a result of the reduction in greenhouse gas (GHGs) emissions into the atmosphere, can be achieved by substituting petroleum-based jet fuel with residual woody biomass-based jet fuel, Fig. 6. Based on the mass allocation method, the GWP reduction results for the ‘100% scenario’ and ‘50% scenario’ are 70.4 and 68.8%, respectively.

The ‘Smog’ and ‘Ecotoxicity’ LCA impact categories also show a net reduction in the environmental impacts when substituting fossil fuel-based bio-jet fuel with woody biomass-based bio-jet fuel. In particular, the ‘Smog’ impact is 26.1 and 4.6% lower than the impact of petroleum-based jet fuel for the 100 and 50% scenarios, respectively. The reduction in the ‘Ecotoxicity’ impact compared to petroleum-based jet fuel is about 79.8% in both scenarios.

The ‘Carcinogenics’ and ‘Respiratory effects’ impact categories are net negative, meaning that the substitution of fossil fuel with biomass-based bio-jet fuel reduces the abundance of carcinogens and various pollutants in the environment that are detrimental to our respiratory health.

The mass allocation-based ‘Acidification’ impact category shows a reduction of 7.0 in the 100% scenario but an increase of 12.9 in the 50% scenario. Similarly, the results for the ‘Eutrophication’ impact category show a reduction of 1.3 in the 100% scenario, but an increase of 15.7 in the 50% scenario.

#### Comparison of fossil fuel-based kerosene and IPK based on system expansion

**Fig. 7:**
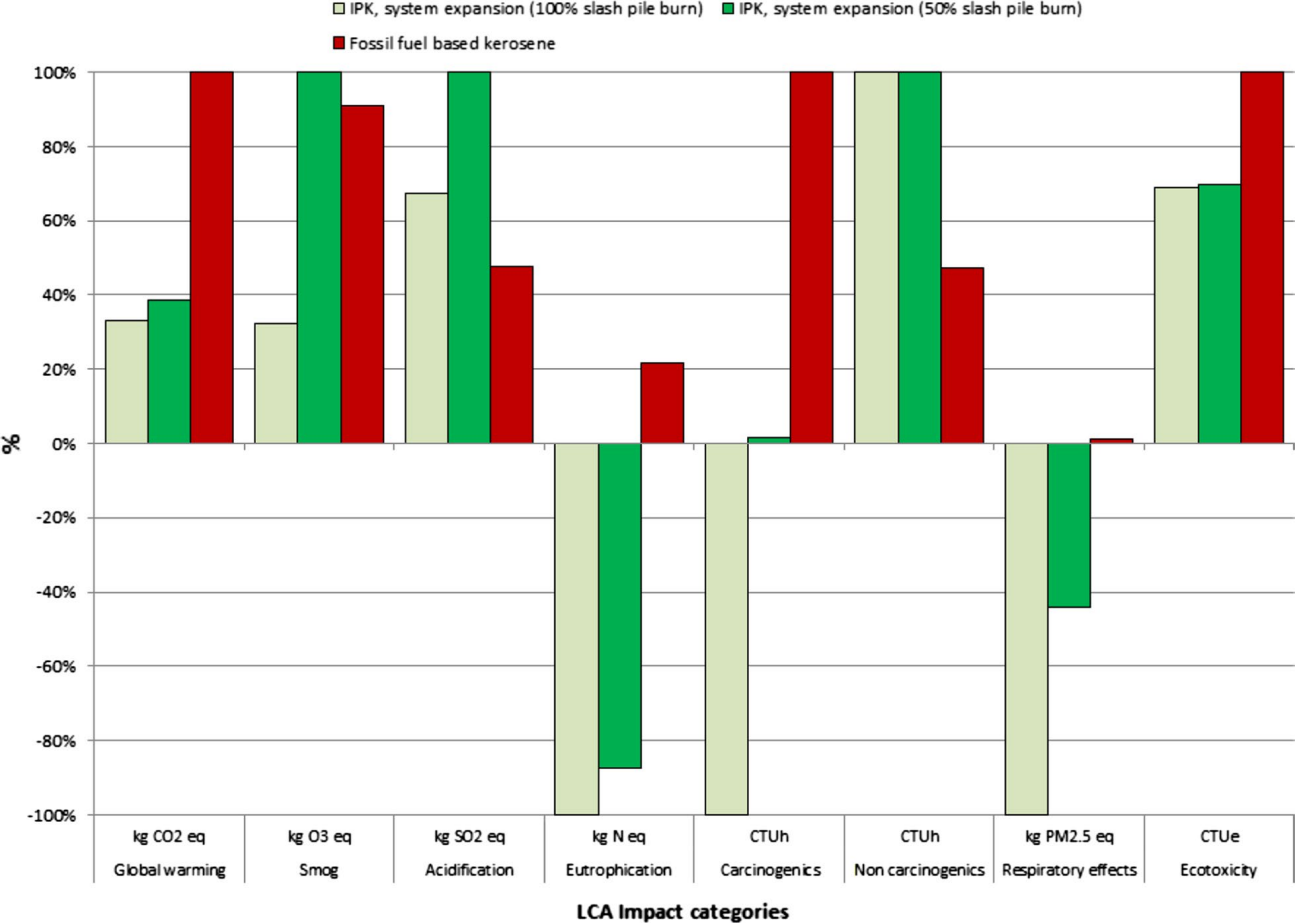
Results of comparative LCA of fossil fuel-based kerosene and IPK based on system expansion (functional unit: 1 GJ)

The results of the comparative LCA obtained using system expansion are represented in Fig. 7. The GWP reduction is 66.9 and 61.3% for the 100 and 50% scenario, respectively.

Apart from GWP, the residual woody biomass-based jet fuel contributes to a substantial reduction in the ‘Smog’ impact category in the 100% scenario (67.6% reduction compared to fossil jet fuel), and in the ‘Ecotoxicity’ impact category (30.9% and 30.4% reduction compared to fossil jet fuel in the 100 and 50% scenarios, respectively), the ‘Eutrophication’, ‘Carcinogenics’, and ‘Respiratory effects’ LCA impact categories are net negative, meaning that the substitution of fossil fuel with biomass-based bio-jet fuel produces a net benefit in these impact categories.

The comparative assessment of impact categories based on the system expansion method shows a more mixed result. Both the 50 and 100% avoided slash pile burn scenarios for the woody biomass-based bio-fuel recorded a higher impact on ‘Acidification’ and ‘Non carcinogenics’ impact categories as compared to its petroleum-based counterpart. In particular, the acidification impact is 41.5 and 110.4% higher than the impact of fossil fuel for the 100 and 50% scenarios, respectively. The ‘Non carcinogenics’ impact in both scenarios is estimated to be about twice that of the corresponding petroleum-based bio-jet fuel impact. The complete results for the comparative LCA using mass allocation and system expansion are presented in Table 4.

**Table 4:** Comparative LCA results using system expansion and mass allocation (functional unit: 1 GJ)

Impact category	Mass allocation		System expansion		Fossil
	IPK (100% slash pile burn)	IPK (50% slash pile burn)	IPK (100% slash pile burn)	IPK (50% slash pile burn	Fossil fuel-based kerosene
Global warming, kg CO_2_ eq	26.00	27.41	29.13	34.00	86.05
Smog, kg O_3_ eq	7.42	9.57	3.57	11.03	10.03
Acidification, kg SO_2_ eq	0.39	0.47	0.59	0.88	0.42
Eutrophication, kg N eq	2.21E−02	2.59E−02	− 1.03E−01	− 9.02E−02	2.24E−02
Carcinogenics, CTUh	− 1.25E−07	5.43E−08	− 6.02E−07	1.94E−08	1.22E−06
Non carcinogenics, CTUh	7.56E−06	7.56E−06	2.51E−05	2.51E−05	1.19E−05
Respiratory effects, kg PM_2.5_ eq	− 1.72E−01	− 6.77E−02	− 6.45E−01	− 2.83E−01	8.54E−03
Ecotoxicity, CTUe	47.25	47.61	161.72	162.97	234.19

### Discussion

The results of the comparative analysis of petroleum-based jet and bio-jet fuel are consistent between the system expansion and mass allocation approaches for the majority of the impact categories. A more than 60% reduction in the global warming potential (which is mandated by EISA) is achieved by substituting petroleum-based jet fuel with residual woody biomass-based jet fuel, independent of the two partitioning methods. Therefore, residual woody biomass represents an environmentally responsible alternative to petroleum for the production of jet fuel. Residual woody biomass presents significant advantages over planted crops or trees, as it does not carry the negative environmental impacts associated with land-use change. As outlined by Budsberg et al. [15], the GWP associated with land-use change can be significant, contributing an additional 12 g CO_2eq_ per MJ of bio-jet fuel used [15]. In the case of IPK, this contribution would add between 35.3 and 46.1% more GWP depending on the scenario. The absence of environmental impacts associated with land-use change gives residual woody biomass-based bio-jet fuels an advantage in terms of environmental performance compared to crop-based alternatives.

In addition to ‘Global warming’, both the partitioning methods reveal net reductions in the ‘Carcinogenics’, ‘Respiratory effects’, and ‘Ecotoxicity’ impact categories. In particular, the ‘Carcinogenics’ and ‘Respiratory effects’ impacts had net negative values (net environmental benefit), as a result of the avoided impact of slash pile burning. This effect can be specifically attributed to the avoided emission of the large amount of PM_2.5_ that is generated during the open burning of woody biomass [57].

However, the results for the ‘Smog’, Acidification’, ‘Non carcinogenics’, and ‘Eutrophication’ impact categories are sensitive to the allocation approach. While the impact categories ‘Smog’ and ‘Acidification’ present different results across the partitioning method adopted but do reveal similar trends, in this study, the major differences in the LCA results are found in the ‘Non carcinogenics’ and ‘Eutrophication’ impact assessment of the bio-fuel across mass allocation and system expansion methods. The ‘Non carcinogenics’ impact is 36.4% lower than that of petroleum-based jet fuel using mass allocation, while it is higher using system expansion. The main contributor to the ‘Non carcinogenics’ impact is the boiler, and allocating the impact of the boiler to the co-products significantly affects the results for this impact category. Using mass allocation, the impact of the boiler is allocated between the co-products based on their mass flow, thus reducing the impact attributable to IPK. As opposed to using system expansion, 100% of the boiler impact is attributed to IPK and no credit is given for the boiler from the avoided impacts of co-products.

The other impact category that is significantly affected by the allocation approach is ‘Eutrophication’. Using the mass allocation approach, the impact on ‘Eutrophication’ is 1.3% lower in the 100% scenario and 15.7% higher in the 50% scenario compared to petroleum-based jet fuel. In contrast, in the system expansion approach, the impact on ‘Eutrophication’ is net negative. This can be directly attributed to the conventional lignosulfonate production process, which imposes a significant impact on ‘Eutrophication’ (Fig. 5). Hence, giving a credit for the displacement of the conventional lignosulfonate in the system expansion process significantly enhances the results for this particular impact category.

It may be noted that the mass flow of lignosulfonate (21,192 kg h^−1^) is almost twice the mass flow of IPK (12,202 kg h^−1^). The relative scale and importance of the co-products limits the applicability of the system expansion approach in this study. This limitation in the applicability of the system expansion approach has been previously reported in other LCA studies dealing with co-products [52]. The system expansion approach can be safely applied in cases where the mass flow of co-products is a small share of the total output. Moreover, the nature of the displaced conventional products, in this case conventional lignosulfonate and conventional activated carbon, also impacts the environmental assessment associated with the primary product under consideration. It should be noted that, in this study, for the impact categories where the conventional activated carbon process has a significant impact, such as ‘Global warming’, ‘Smog’, ‘Acidification’, and ‘Respiratory Effect’ (Fig. 5), the application of both the mass allocation approach and the system expansion approach produces consistent results. However, when performing an LCA of bio-fuels, where nonfuel products are a large share of the total output, the method generates misleading results for the primary product, in this case bio-jet fuel. Therefore, as outlined by Wang et al. while the system expansion is generally advocated for conducting LCAs, when the mass flows of co-products are very different, system expansion may not be appropriate at all for the LCA of the fuel product and other allocation methods should be considered [52].

## Conclusions

In this study, a ‘cradle-to-grave’ life cycle assessment, using a woods-to-wake system boundary, was performed for residual lignocellulosic biomass-based bio-jet fuel, produced alongside two co-products ‘activated carbon’ and ‘lignosulfonate’. Two different approaches were used to deal with the co-products, system expansion and mass allocation. Although avoiding allocation by expanding the system boundary is the recommended approach by international standards, in this study, the relative scale and importance of the co-products limit the applicability of the system expansion approach.

Independent of the partition approach, comparing the results of the ‘cradle-to-grave’ life cycle assessment of IPK with the ‘cradle-to-grave’ life cycle assessment of petroleum-based jet fuel, more than 60% reduction in the global warming potential is achieved, exceeding the US Environmental Protection Agency mandate for GWP reduction.

The residual woody biomass-based jet fuel also contributes to a substantial reduction in the ‘Smog’, ‘Carcinogenics’, ‘Respiratory effects’, and ‘Ecotoxicity’ impact categories. In particular, the ‘Carcinogenics’ and ‘Respiratory effects’ impact resulted in net negative values (net environmental benefit), as a result of the avoided impact of slash pile burning.

Overall, the residual woody biomass recovered from slash piles represents a valuable alternative to petroleum to produce jet fuel with a lower impact on global warming and net reduction in local air pollution. However, the production of woody biomass-based bio-jet fuel did not show any significant improvement in the ‘Acidification’ and ‘Eutrophication’ impact categories. Future research should focus on the optimization of the chemical processes of the bio-refineries to reduce the impacts on these categories.

## Abbreviations

AC: activated carbon
ATJ: alcohol-to-jet
COD: chemical oxygen demand
EISA: energy independence and security act
FRS: fermentation residual stillage
GHG: greenhouse gas
GREET: greenhouse gases, regulated emissions, and energy use in transportation
GWP: global warming potential
IPK: iso-paraffinic kerosene
LCA: life cycle assessment
LCIA: life cycle impact assessment
LS: lignosulfonate
LS 50%: 50% concentrated lignosulfonate
PM: particulate matter
PNW: Pacific Northwest
SPORL: sulfite pretreatment to overcome recalcitrant of lignocellulose
SSL: spent sulfite liquor
SVOC: semi-volatile organic compounds
TRACI: tool for reduction and assessment of chemicals and other environmental impacts
VOC: volatile organic compounds
WoTW: woods-to-wake
WTW: well-to-wake
